# Effects of heterologous expression of phosphoenolpyruvate carboxykinase and phosphoenolpyruvate carboxylase on organic acid production in *Aspergillus carbonarius*

**DOI:** 10.1007/s10295-015-1688-4

**Published:** 2015-09-24

**Authors:** Lei Yang, Mette Lübeck, Peter S. Lübeck

**Affiliations:** Section for Sustainable Biotechnology, Department of Chemistry and Bioscience, Aalborg University Copenhagen, A. C. Meyers Vaenge 15, 2450 Copenhagen SV, Denmark

**Keywords:** *Aspergillus carbonarius*, Phosphoenolpyruvate carboxykinase, Phosphoenolpyruvate carboxylase, Citric acid, Organic acids

## Abstract

*Aspergillus carbonarius* has a potential as a cell factory for production of various organic acids. In this study, the organic acid profile of *A. carbonarius* was investigated under different cultivation conditions. Moreover, two heterologous genes, *pepck* and *ppc*, which encode phosphoenolpyruvate carboxykinase in *Actinobacillus succinogenes* and phosphoenolpyruvate carboxylase in *Escherichia coli,* were inserted individually and in combination in *A. carbonarius* to enhance the carbon flux toward the reductive TCA branch. Results of transcription analysis and measurement of enzyme activities of phosphoenolpyruvate carboxykinase and phosphoenolpyruvate carboxylase in the corresponding single and double transformants demonstrated that the two heterologous genes were successfully expressed in *A. carbonarius.* The production of citric acid increased in all the transformants in both glucose- and xylose-based media at pH higher than 3 but did not increase in the pH non-buffered cultivation compared with the wild type.

**Electronic supplementary material:**

The online version of this article (doi:10.1007/s10295-015-1688-4) contains supplementary material, which is available to authorized users.

## Introduction

Organic acid production by filamentous fungi has been investigated for decades. The applications of organic acids, especially carboxylic acids, can be found in a wide range of industries including food, beverage, cosmetics, pharmaceuticals, and detergents [31]. Presently, some organic acids, such as citric acid, gluconic acid, and lactic acid, can be produced via biological processes using one-step fermentation [21, 28, 35]. Certain strains of *Aspergillusniger* are used commercially for the production of citric acid due to high titer of citric acid (>150 g/L) combined with utilization of cheap substrates [24, 34]. However, some other organic acids with huge marketing potential, like malic acid and fumaric acid, are still produced via chemical processes at relatively low cost compared with biological processes [9, 10, 13]. Suitable producing strains are considered as the key factors influencing the economic feasibility of the entire biological processes for production of organic acids. The black filamentous fungus *Aspergilluscarbonarius* can naturally produce high amount of citric acid in response to stress conditions using a variety of substrates including hexoses and pentoses [7, 36]. For the last decade, research carried out on *A. carbonarius* has mainly focused on the food contamination caused by its mycotoxin ochratoxin A (OTA), which is normally considered as an important issue for microorganisms to be applied in industrial processes. However, a recent report published on *A. carbonarius* ITEM 5010 revealed a new insight into the OTA biosynthetic pathway and demonstrated an efficient way to eliminate the OTA production through genetic engineering [6]. The progress in inactivation of OTA biosynthetic pathway paves the way to industrial applications of *A. carbonarius*. In addition, it is reported that *A. carbonarius* is able to produce a series of hydrocarbons from different types of lignocellulosic materials [32]. The abilities of *A. carbonarius* to produce various types of organic acids and utilize a wide range of carbon sources indicate its potential as a cell factory for industrial production of organic acids using renewable biomass (e.g., lignocellulosic materials) [32, 37]. *A. carbonarius* is phylogenetically close to *A. niger,* resembling many features in the morphology and physiology [1], which provides the possibility to use *A*. *niger* as a reference strain for investigating acid producing pathways in *A.carbonarius*. However, the differences in fungal metabolism between two fungal species are not negligible.

In filamentous fungi, the reductive tricarboxylic acid (rTCA) branch is highly involved in the synthesis of several organic acids including malic acid, fumaric acid, and citric acid [3, 19, 39]. In *Aspergillusflavus*, a sharply increased enzyme activity of pyruvate carboxylase, which carries out the conversion of pyruvate to oxaloacetate (OAA), was detected while malic acid was accumulated during cultivation [27]. In *Rhizopus oryzae*, fumaric acid is mainly produced through the rTCA branch involving the conversion of oxaloacetate to malate catalyzed by malate dehydrogenase and dehydration of malate to fumarate catalyzed by fumarase [17]. For *A. niger*, it has been shown that genetic modifications in the rTCA branch exhibited effects on citric acid production [3]. Citric acid production was initiated in *A. niger* in the early phase of cultivation when intracellular concentration of malate was increased by overexpressing cytosolic malate dehydrogenase, and enhanced citric acid production was observed when fumarase was overexpressed to increase the concentration of fumarate in the cytosol. Those effects on citric acid production in *A. niger* might result from an anti-port between dicarboxylic acids and citric acid across the mitochondrial membrane [3, 16], similar to what was elucidated in *Saccharomyces cerevisiae* [30].

**Fig. 1:**
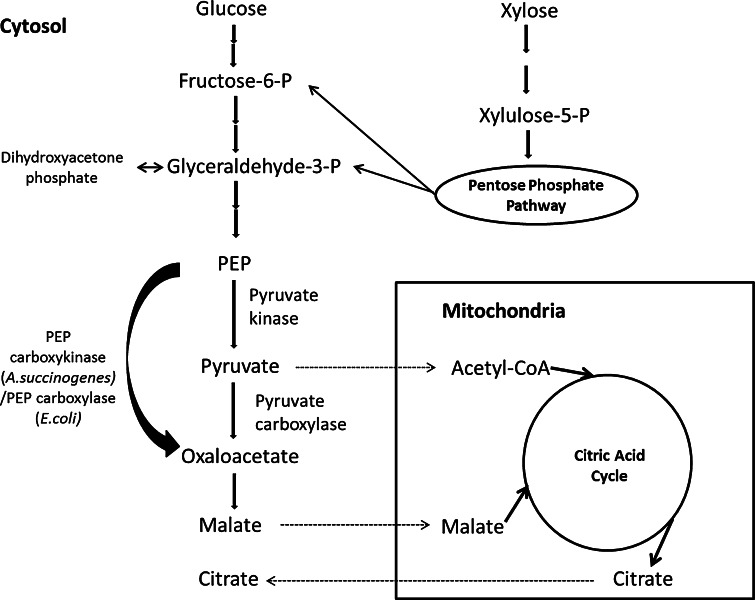
Proposed metabolic pathway for organic acid production in *A. carbonarius*. The *bold arrow* indicates the introduced pathway; the *dash arrow* indicates transport across mitochondrial membrane; and *multiple arrows* indicate the multiple reactions omitted in the pathway map

In this study, an alternative cytosolic pathway carrying out one-step conversion of phosphoenolpyruvate (PEP) to oxaloacetate was inserted into *A. carbonarius* to enhance the carbon flux toward oxaloacetate as the onset of the rTCA branch (Fig. 1). Two exogenous genes *pepck* and *ppc* encoding phosphoenolpyruvate carboxykinase (AsPEPCK from *Actinobacillus succinogenes*) and phosphoenolpyruvate carboxylase (EcPPC from *Escherichia coli*), which can convert PEP to oxaloacetate with a fixation of CO_2_, were expressed in *A. carbonarius.* The EcPPC carries out an irreversible conversion of PEP to oxaloacetate and one phosphate group [8]. The AsPEPCK carries out a reversible conversion of PEP to oxaloacetate with the generation of one ATP. Commonly in microorganisms, PEPCK is responsible for converting oxaloacetate to PEP in the gluconeogenic pathway, but there is no phosphoenolpyruvate carboxylase activity exhibited in *A. succinogenes*, and PEPCK is used to produce oxaloacetate directly from PEP in this species [18, 22]. It has been reported that overexpression of EcPPC in *R. oryzae* resulted in elevated fumaric acid production indicating its impact on organic acid production via the rTCA branch [39]; however, there is yet no report regarding expression of AsPEPCK in filamentous fungi. In this study, the aims were to express both genes in *A. carbonarius*, individually, and in combination, and to compare the impacts of the genetic modifications on organic acid production in cultivations using different conditions. Besides, a preliminary analysis on the organic acid profile of *A. carbonarius* wild type was conducted at different pH to investigate the impact of pH change on organic acid production.

## Materials and methods

### Strains

*Aspergillus carbonarius* ITEM5010 (ATCC^®^ MYA-4641™) was selected to construct new strains. *E. coli* K-12 (ATCC^®^ 10798™) and *A. succinogenes* 130Z (ATCC^®^ 55618™) were used to obtain the genes *ppc* and *pepck*, respectively, for the genetic modifications.

### Culture media

Fungal strains were cultured in potato dextrose agar (PDA) medium at 30 °C for harvesting of spores. For genomic DNA extraction, the strains were cultured in yeast extract peptone dextrose (YPD) medium containing (g/L) yeast extract, 10; peptone, 20; and glucose, 20, at 30 °C for 2 days after which mycelia were collected for DNA isolation. *E. coli* K-12 was cultivated in LB medium composed of (g/L) tryptone, 10; yeast extract, 5; and NaCl, 10 at 37 °C, and *A. succinogenes* 130Z was grown in BHI (Brain Heart infusion) medium containing 15 g/L brain heart infusion powder (Sigma) at 37 °C. The pre-culture was carried out in the medium containing (g/L): yeast extract, 3.6 and peptone, 10. The cultivation was carried out in the three different production media for organic acid production. For buffered pH conditions, the glucose production medium consisted of (g/L) glucose, 100; (NH_4_)_2_SO_4_, 2; KH_2_PO_4_, 0.15; K_2_HPO_4_, 0.15; MgSO_4_·7H_2_O, 0.1; CaCl_2_·2H_2_O, 0.1; NaCl, 0.005; ZnSO4, 0.1 g/L; FeSO_4_ 7H_2_O, 0.005; and CaCO_3_, 60 [26]. For pH non-buffered conditions, the glucose production medium was made using the above-mentioned recipe but omitting calcium carbonate. The xylose production medium contains (g/L) d-xylose, 100; NH_4_NO_3_, 2.5; K_2_HPO_4_, 0.1; MgSO_4_·7H_2_O, 1; CaCl_2_·2H_2_O, 0.168; KCl, 0.43; ZnSO_4_·7H_2_O, 4.5 × 10^−3^; and FeSO_4_·7H2O, 0.75 × 10^−3^ [36].

### Construction of plasmids expressing *ppc* and *pepck*

Genomic DNA isolated from *E. coli* K-12 and *A. succinogenes* 130Z was used to amplify the genes *ppc* and *pepck,* respectively, with primers containing uracil overhangs compatible with the USER cloning-based plasmid pSBe1 [12]. The PCR reactions amplifying *ppc* (~2.6 kb) and *pepck* (~1.6 kb) were set up in 50 μL reaction volumes containing 5 μL 10× Pfu turbo cx reaction buffer; 1 μL 10 μM dNTP; 2.5 μL 10 μM forward primer and 2.5 μL 10 μM reverse primer (Table 1); 1 μL Pfu turbo cx polymerase (Agilent); appropriate amount of DNA template; and water added up to 50 μL. The cycling parameters in the PCR program were initial denature step at 95 °C for 3 min; 25–30 cycles of denature step at 94 °C for 30 s; annealing step at 55–65 °C for 30 s; elongation step at 72 °C for a specific amount of time calculated by the size of desired fragments (1 min/kb); and followed by a final elongation step at 72 °C for 5 min. The *ppc* and *pepck* genes were then cloned separately into the vector pSBe1 between the *gpdA* promoter and the *TrpC* terminator as described previously [12] followed by transformation of *E. coli* DH5α which was grown in LB medium with 100 μg/ml ampicillin at 37 °C (adding 15 g/L agar for solid LB plate). The plasmids pSBe1ppc and pSBe1pepck were verified in colony PCR and used for fungal transformation. To construct double transformants with two genes inserted (*pepck* and *ppc*), the two plasmids were used together in co-transformation of protoplasts. In order to increase the frequency of co-transformation, the hygromycin resistance gene *hph* was inactivated in the plasmid pSBe1pepck by linearizing the plasmid through *NcoI* restriction site which solely exists in the region of *hph* gene in the plasmid (Supplementary material 2). The sequences, design of primers, and plasmid maps were handled using the CLC workbench (CLC Bio).

**Table 1:** Primers used in this research

Name	Sequence (5′ → 3′)	Annotation
Pck uFw1	AGAGCGAUATGACTGACTTAAACAAACTCGTT	USER cloning of *pepck*
Pck uRv1	TCTGCGAUTTATGCTTTTGGACCGGCGCCA	USER cloning of *pepck*
Ppc uFw1	AGAGCGAU ATGAACGAACAATATTCCGCA	USER cloning of *ppc*
Ppc uRv1	TCTGCGAU AGATTAGCCGGTATTACGCAT	USER cloning of *ppc*
Pck Fw1	GCTTAAGAATGCCGCACCGAA	PCR of *pepck* on cDNA
Pck Rv1	TTATGCTTTTGGACCGGCGCCA	PCR of *pepck* on cDNA
Ppc Fw1	CGATTGCCAACGATTCCCAT	PCR of *ppc* on cDNA
Ppc Rv1	AACTCGGCCTGCAATACGTTC	PCR of* ppc* on cDNA
Actin Fw	AGAGCGGTGGTATCCATGAG	PCR of *beta-actin* on cDNA
Actin Rv	TGGAAGAGGGAGCAAGAGCG	PCR of *beta-actin* on cDNA

### Protoplast transformation

Protoplasts of *A. carbonarius* were made from young mycelia as previously described [11]. The final concentration of protoplasts for aliquots was adjusted to 2 × 10^7^/mL, and the fresh protoplasts were preserved at −80 °C with the addition of 40 % PEG4000 and 7 % DMSO. The procedure for protoplast transformation was described in [37]. 5 μg of plasmids in 10 μL was added into 100 μL protoplast suspension. For co-transformation, 4 μg linearized plasmid pSBe1pepck and 1 μg circular plasmid pSBe1ppc in total volume of 10 μL were added into protoplast suspension. Transformants were selected after sporulation. Single colonies of individual transformants were obtained by streaking out the spores on PDA medium followed by overnight incubation at 30 °C. To prepare genomic DNA, the spores of the selected transformants were inoculated into YPD medium and grown at 30 °C for 24 h, and young mycelia were used directly in genomic DNA extraction the following day. The inserted target genes in the transformants were verified by examining the amplified fragments with expected sizes in PCR. The verified transformants were then preserved for further steps.

### RNA extraction and transcription analysis of inserted genes

The mycelia of transformants were harvested after 3 days cultivation in YPD medium and directly used for RNA extraction. RNA was extracted using total RNA isolation kit (A&A Biotechnology) according to the procedure described by the manufacturer, and the purified RNA samples were treated with DNase to remove potential contaminating DNA by incubating approx. 1 μg RNA with 1 unit DNaseI (Fermentas) and 1 μL 10× reaction buffer in total 10 μL reaction volume at 37 °C for at least 30 min. The reaction was terminated by adding 1 μL 50 mM EDTA and incubated at 65 °C for 10 min. The treated RNA samples were used directly to make cDNA without any further purification.

The cDNA was generated using the reverse transcription kit (Bio-rad). The reaction setup was made as follows: 1 μg of RNA template was mixed with 1 μL transcriptase and 4 μL reaction mix containing the random hexamers in total 20 μL reaction volume. The reaction mix was then incubated at 25 °C for 5 min; 42 °C for 30 min; and 85 °C for 5 min. The cDNA product was applied to PCR without clean-up. The transcription of the inserted gene in the transformants was verified by amplifying a partial sequence of the inserted genes (150–200 bp) from cDNA in PCR. A housekeeping gene, *beta*-*actin*, was used as control to confirm the quality of cDNA and preliminarily analyze the transcription levels of the inserted gene by comparing the intensity of amplified bands in the gel electrophoresis. The PCR reactions were set up in 50 μL reaction volumes containing 5 μL 10× RUN buffer; 1 μL 10 μM dNTP; 2.5 μL 10 μM forward primer and 2.5 μL 10 μM reverse primer (Table 1); 1 μL RUN polymerase (A&A Biotechnology); cDNA template; and water added up to 50 μL. The cycling parameters in the PCR program were initial denature step at 95 °C for 3 min; 25 cycles of denature step at 94 °C for 30 s; annealing step at 55–65 °C for 30 s; elongation step at 72 °C for a specific amount of time calculated by the size of desired fragments (1 min/kb); and followed by a final elongation step at 72 °C for 5 min.

### Enzyme activity assay

*Aspergillus carbonarius* wild type and transformants were cultivated in YPD medium for 24 h at 30 °C. The mycelium was then harvested and washed with 0.01 M Tris–HCl buffer before suspension in 0.01 M Tris–HCl buffer supplemented with 0.2 M KCl. The cell extract was prepared by treating the mycelia suspension with glass bead beaters for 2 min in a Fast Prep^®^-24 instrument (MP Biomedicals) followed by centrifugation at 20.000×*g* for 10 min. The supernatant was used directly for enzyme assay [25].

All the enzymes were assayed spectrophotometrically using the Spectrophotometer DR3800 (Hach company) at room temperature in 1.5-mL cuvettes (1.0 cm light path). The PPC activity was measured in a reaction solution containing 0.1 M Tris–HCl buffer at pH 8.0, 0.01 M MgCl_2_, 2.5 mM phosphoenolpyruvic acid, 0.2 mM NADH, 0.01 M NaHCO_3_, and 5 units of malate dehydrogenase [39]. The reaction was started by adding cell extracts containing 0.4 mg protein, and the rate of decrease in absorbance of NADH at 340 nm was measured. The extinction coefficient was 6.22 mM^−^/cm. The specific enzyme activity was calculated as the oxidation of 1 μmol NADH based on the amount of protein per minute at 30 °C and pH 8.0. For measuring enzyme activity of AsPEPCK toward carboxylation of PEP, 2.5 mM ADP was added into the reaction mixture with the same assay procedure as described above for EcPPC. The enzyme activity of AsPEPCK in double transformants was obtained by subtracting the enzyme activity of EcPPC from the total enzyme activity measured with PEPCK enzyme assay in the same sample. The concentration of protein in cell extract was measured by using BCA protein assay reagent kit (Thermo Scientific). The increased fold of total enzyme activities of PEP carboxylation in the selected transformants was calculated by summing up measured activities of PEPCK and PPC in the individual strains and comparing the activity of selected transformants to the wild type.

### Organic acid production

Spores of fungal strains were harvested from PDA medium after 5–7 days of cultivation at 30 °C. The spores were collected by filtering through sterilized Miracloth (Fisher Scientific) to remove the mycelium and counted in a hemocytometer followed by inoculation into 50-mL falcon tubes containing 10 mL pre-culture medium. The final concentration of spores in the pre-culture medium was approximately 1 × 10^5^/mL. The pre-cultivation was carried out at 30 °C with agitation at 250 rpm for 2 days. Pellets formed in the pre-cultivation were filtered through sterile miracloth and added to 20 mL production media in 100-mL flasks and incubated 30 °C with agitation at 180 rpm for 7 days. The initial pH of the production media was adjusted to 5.5 before inoculation. All the cultivations were carried out in triplicates.

### Analysis of extracellular metabolites

The samples taken from the cultivation broth were acidified with 72 % sulfuric acid to precipitate the calcium ion in form of calcium sulfate and exchange the organic acid back to liquid phase. The acidified samples were incubated at 80 °C for at least 15 min to complete the reaction followed by centrifugation at 14,000 rpm for 1 min. HPLC analysis of the supernatants for sugar (glucose and xylose) and organic acids was carried out in an Aminex 87H column (Bio-rad) at 60 °C by using HPLC mobile phase at a flow rate of 0.6 mL/min. The HPLC samples were kept at 4 °C in the machine during the analysis process and then stored at −20 °C. Besides HPLC analysis, measurements of l-malic acid and d-gluconate in the samples were carried out using l-malate (l-malic acid) kit and d-gluconate kit (Megazyme).

### Fungal biomass measurement

To measure the dry weight of fungal biomass obtained in non-buffered pH conditions, the fungal culture was filtered through the filter paper (Whatman) and then washed thoroughly with distilled water until pH reached 6.0. The washed fungal cells on the filter paper were dried at 100 °C for 48 h before weighing. The measurement of fungal biomass obtained in pH buffered condition was carried out in the same procedure as described before except an additional acidification step. The fungal culture was acidified with 1 N HCl solution to dissolve calcium carbonate before the washing step with distilled water.

### Statistical analysis

Unless specified, all the experiments were carried out in triplicates, and the average value with standard deviation is reported. *T* test analysis was performed to evaluate the significant difference in the reported results (*p* = 0.05 as the threshold of significant difference).

## Results

### Analysis of extracellular organic acids produced by wild type *A. carbonarius* under non-buffered and buffered pH conditions

**Fig. 2:**
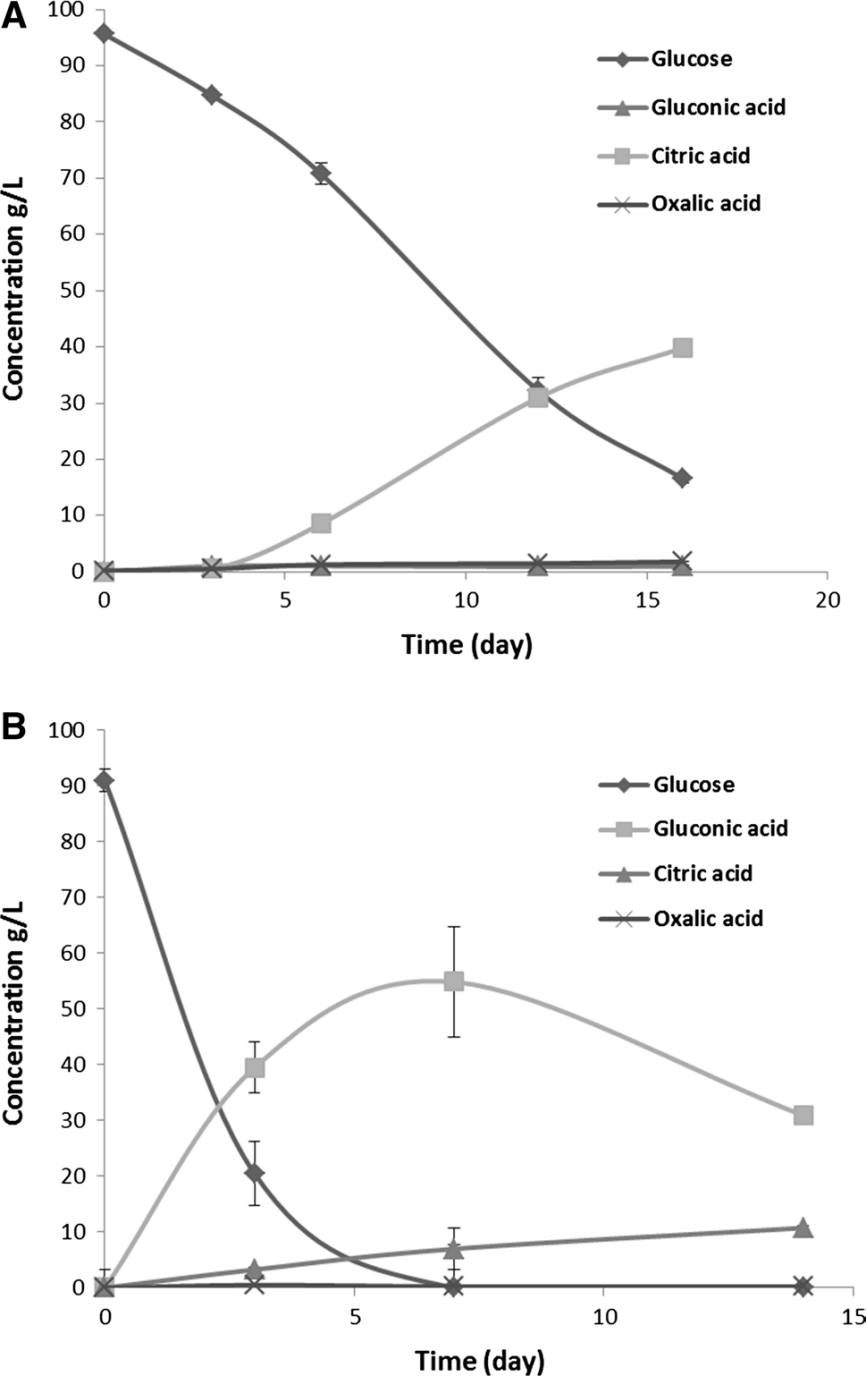
Glucose consumption and production of major extracellular organic acids. **a** concentration of measured organic acids (g/L) at non-buffered pH (error bars are presented in the curves but may be invisible due to the size); **b** concentration of measured organic acids (g/L) at buffered pH 5.5. (in shake flasks, 180 rpm, at 30 °C)

In order to analyze organic acid production by *A. carbonarius* wild type strain, cultivation was carried out in shake flasks in non-buffered pH and buffered pH 5.5 conditions in glucose-based media. At non-buffered pH, *A. carbonarius* produced approx. 40 g/L citric acid (Fig. 2a) as the main extracellular organic acid and low amount of oxalic acid (1.8 g/L). During the cultivation, the pH decreased to 2–2.5 within 3 days and stayed at this level for the rest of the period of cultivation (Supplementary material 1). During cultivation at pH 5.5, which was maintained by addition of CaCO_3_, the pattern of extracellular organic acids was different. Instead of accumulating citric acid as the main organic acid, high amount of gluconic acid was produced due to the direct conversion of glucose catalyzed by glucose oxidase, which also led to a rapid depletion of glucose as carbon substrate after the first 7 days (Fig. 2b). A shift of carbon source from glucose to gluconate occurred in the later phase of cultivation, and this shift resulted in a continued production of citric acid. In addition, malic acid was present at a concentration of 0.06 g/L in these conditions on day 7, which indicated that *A. carbonarius* was capable of transiently producing malate during the cultivation (data not shown). However, malic acid was not detected in measurable quantity in non-buffered pH cultivation.

### Insertion of gene *pepck* and *ppc* in *A. carbonarius* by protoplast transformation

The *ppc* and *pepck* genes were amplified from *E. coli* K-*12* and *A. succinogenes* 130Z, respectively, and then cloned into vector pSBe1 for heterologous expression in *A. carbonarius* wild type strain. After inserting the target genes into the vector, the resulting plasmids, pSBe1ppc and pSBe1pepck (Supplementary material 2), were used in protoplast transformation of *A. carbonarius*. For co-transformation, the plasmid pSBe1pepck was linearized at the *NcoI* restriction site to inactivate the *hph* gene and was used for transformation of *A. carbonarius* together with plasmid pSBe1ppc. Transformants with inserted *hph* gene were able to grow on minimal medium with 100 µg/mL hygromycin. Nine stable transformants with the *pepck* gene, ten transformants with the *ppc* gene, and three double transformants with both genes inserted were obtained. All transformants were screened for organic acid production in glucose-based medium at pH 5.5 (data not shown). The best organic acid-producing transformants of each type of genetic modification were selected for further analysis.

### Transcription analysis of gene *pepck* and *ppc*

**Fig. 3:**
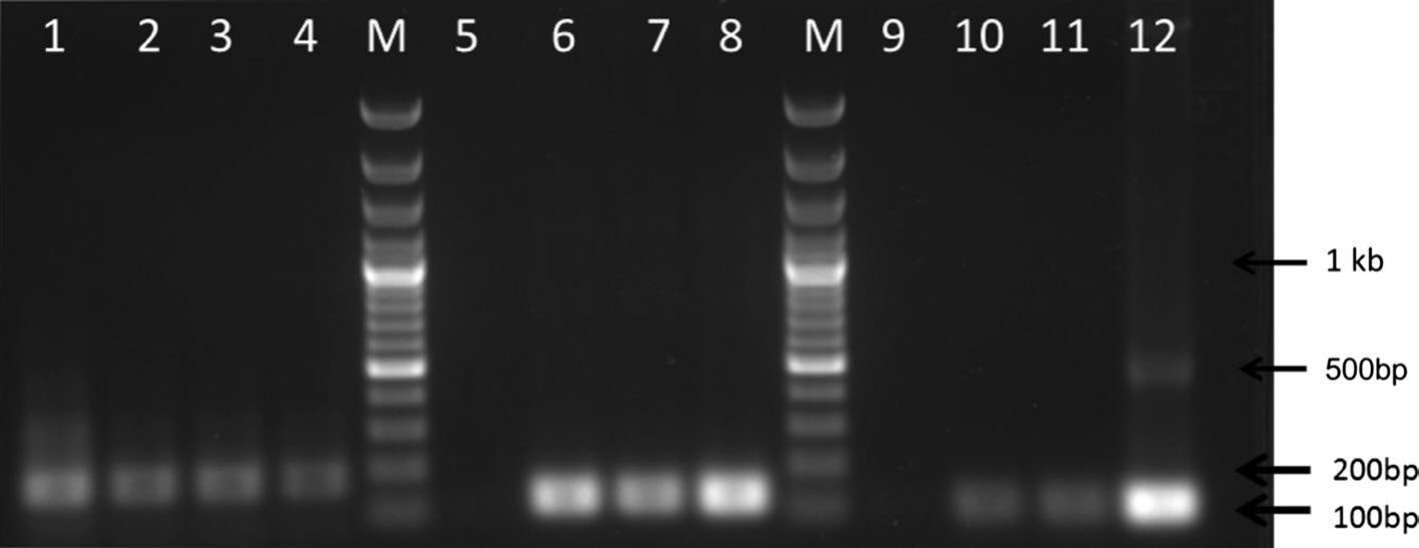
PCR verification of the transcription of *ppc* and *pepck* genes using cDNA as template. Amplification of *beta-actin* DNA (approx. 180 bp): *lane 1* wild type; *lane 2* *pepck* transformant; *3* *ppc* transformant; *4* double transformant; Amplification of *pepck* gene (approx. 150 bp): *lane 5* wild type; *lane 6* *pepck* transformant; *lane 7* double transformant; *lane 8* positive control (plasmid pSBe1pepck); Amplification of *ppc* gene (approx. 140 bp): *lane 9* wild type; *lane 10* *ppc* transformant; *lane 11* double transformant; *lane 12* positive control (plasmid pSBe1ppc). The appearance of a smear band might be caused by overloaded DNA templates

In order to verify the expression of the inserted genes in the derived strains, cDNA was synthesized from purified RNA of the three selected transformants and then used as templates in PCR to amplify partial sequences of target genes. As shown in Fig. 3, expression of *pepck* and *ppc* was verified by PCR. As a control for the quality of the cDNA, PCR amplification of beta-actin on the cDNA was carried out.

### Enzyme assays

**Fig. 4:**
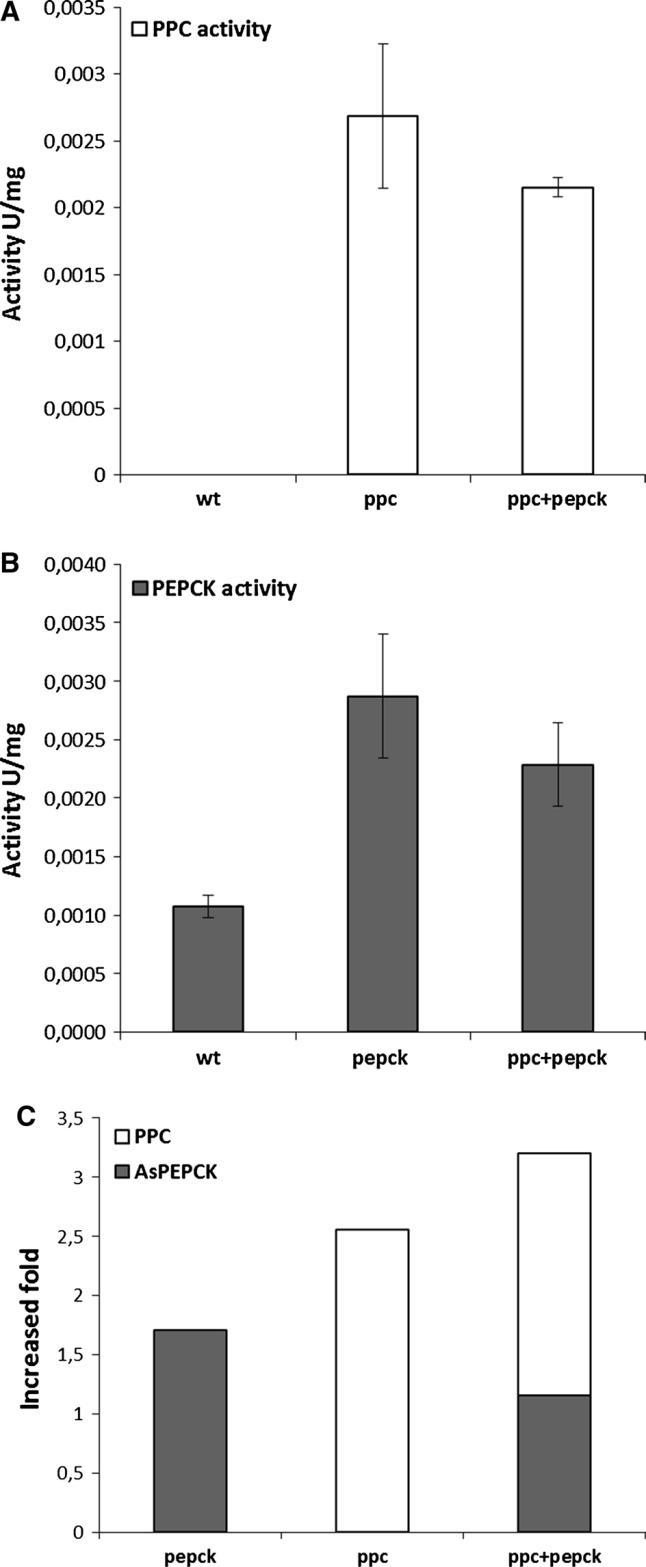
Enzyme activity measurements in the wild type and the selected transformants. **a** PPC activities (U/mg) in the wild type, *ppc* transformant, and double transformants; **b** PEPCK activities (U/mg) in the wild type, *pepck* transformant, and double transformants; **c** Estimated increased fold of enzyme activity contributed by the two genes *pepck* and *ppc* toward PEP carboxylation in transformants compared to the wild type (calculated results without standard deviations)

The enzyme activities of PEP carboxylase (EcPPC) and PEP carboxykinase (AsPEPCK) were measured in *A. carbonarius* wild type and in the selected transformants. As shown in Fig. 4a, the EcPPC activity was detected in cell extract of single and double transformants containing the *ppc* gene but not in the wild type. Since no PPC activity has been reported to occur in filamentous fungi, the absence of PPC activity in the wild type of *A. carbonarius* was expected. However, the specific enzyme activity of AsPEPCK in the transformants could not be compared in a similar way as activity of native PEPCK in *A. carbonarius* was also present. The enzyme activity of the inserted AsPEPCK was measured by comparing the increase of PEPCK activity of the transformants compared to the wild type. The activity of AsPEPCK in the double transformant was obtained by subtracting the measured EcPPC activity from total enzyme activity since the enzyme activity of PEPCK and EcPPC could not be distinguished in the PEPCK enzyme assay. As shown in Fig. 4b, a significant increase in PEPCK activity was measured in both single and double transformants compared with the wild type (0.0029 U/mg in *pepck* transformant and 0.0023 U/mg in double transformant, vs. 0.0011 U/mg in the wild type, *p* < 0.05), which is an indication of expression of AsPEPCK in the transformants. Due to the insertion of the two genes, the enzyme activity of PEP carboxylation was increased approx. 1.7 times, 2.5 times, and 3.2 times in *pepck* transformant*, ppc* transformant, and double transformant, respectively, compared to the wild type.

### Effects of inserting *ppc* and *pepck* on organic acid production at non-buffered pH in glucose medium

**Fig. 5:**
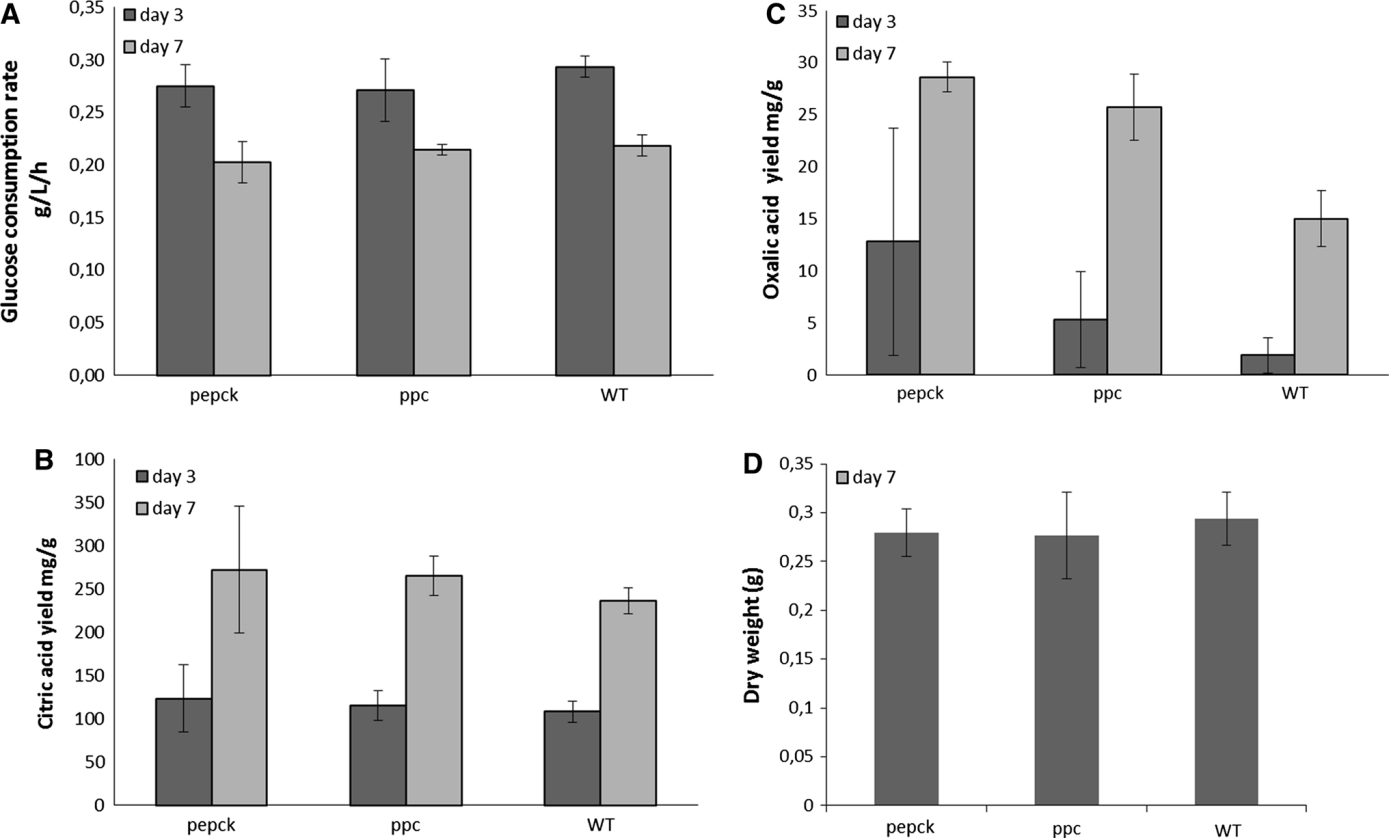
Glucose consumption rate and major extracellular organic acids in non-buffered pH glucose medium. **a** glucose consumption rate (g/L/h) by the wild type and selected transformants; **b** citric acid yield (mg/g glucose) in the wild type strain and selected transformants; **c** oxalic acid yield (mg/g glucose) in the wild type strain and selected transformants; **d** dry weight (g) of fungal biomass after 7 days cultivation. (In shake flasks, 180 rpm, at 30 °C)

In order to investigate the effects of the inserted *ppc* and *pepck* genes on organic acid production, especially citric acid production, the two single transformants were tested in shake flasks in glucose medium without pH control. During the cultivation, pH decreased gradually along with the organic acid production by the two transformants and the wild type and reached a pH at 2–2.5 within 3 days (Supplementary material 3). As shown in Fig. 5a and b, on both day 3 and 7, there was no significant difference in citric acid yield and glucose consumption rate by any of the strains. A minor increase in oxalic acid yield was observed in the derived strains compared to the wild type (Fig. 5c). All the strains produced a similar dry weight after 7 days cultivation (Fig. 5d).

### Effects of inserting *ppc* and *pepck* on organic acid production at buffered pH in glucose medium

**Fig. 6:**
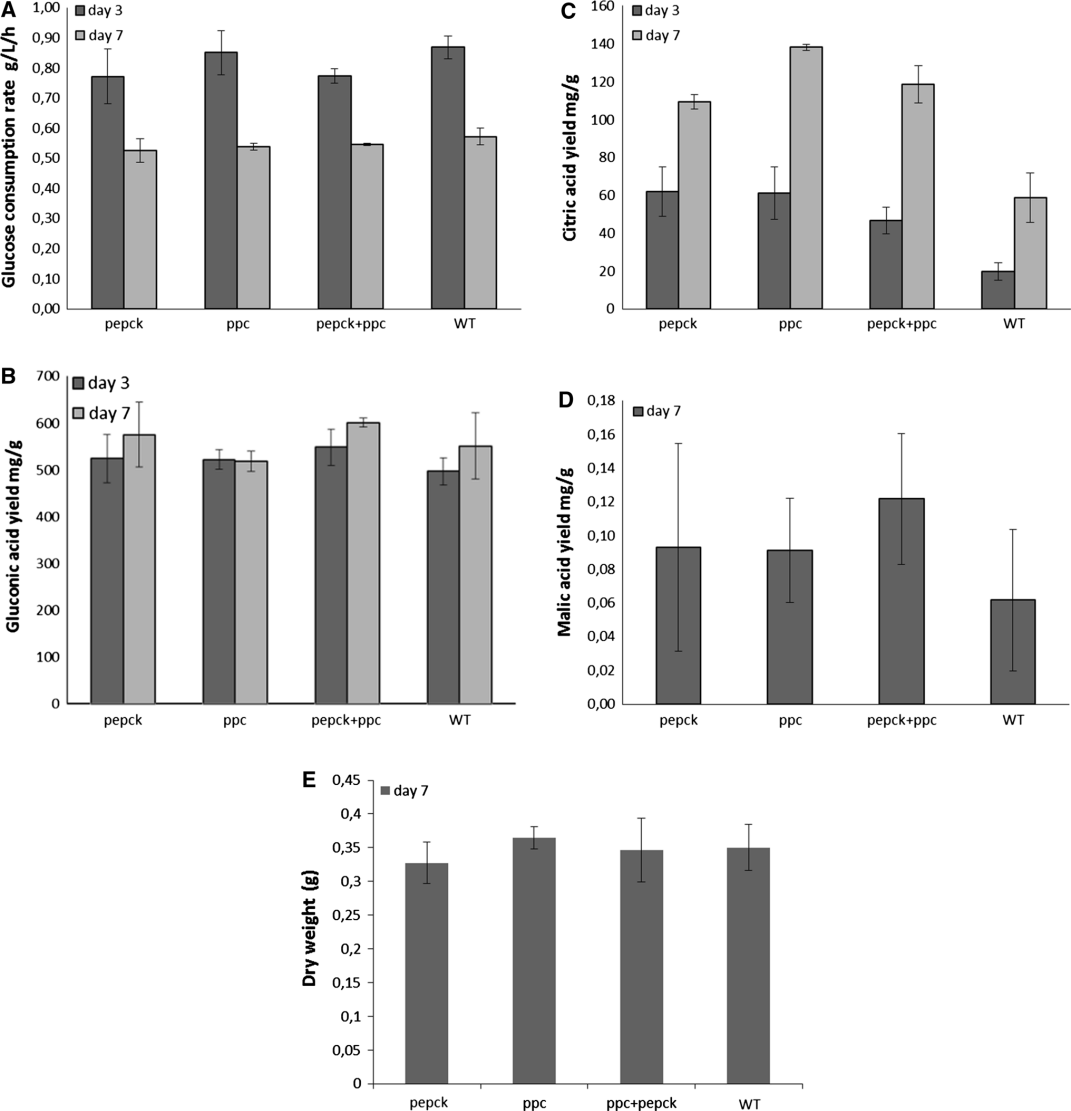
Glucose consumption rate and major extracellular organic acids in buffered pH (5.5) glucose medium. **a** glucose consumption rate (g/L/h) by the wild type and selected transformants; **b** gluconic acid yield (mg/g glucose) in the wild type strain and selected transformants; **c** citric acid yield (mg/g glucose) in the wild type strain and selected transformants; **d** malic acid yield (mg/g glucose) in the wild type strain and selected transformants; **e** dry weight (g) of fungal biomass after 7 days cultivation. (In shake flasks, 180 rpm, at 30 °C)

In order to investigate the effects of the inserted *ppc* and *pepck* genes on organic acid production, especially citric acid production, the single and the double transformants were tested in shake flasks in glucose medium for 7 days at pH 5.5. At this pH, the yield of citric acid was significantly increased in all the selected transformants compared with the wild type (Fig. 6c). After 7 days of cultivation, the citric acid yield of the wild type was 58 mg/g (mg citric acid/g glucose), whereas the *pepck* and *ppc* transformants with the single genetic modifications and the double transformant with two genetic modifications produced 109, 138, and 118 mg/g citric acid, respectively (*p* < 0.05). A slight increase of malic acid production was also observed in the derived strains (Fig. 6d). However, the yield of gluconic acid, biomass growth, and glucose consumption rate were not affected by the genetic modifications in any of the transformants compared with the wild type after 7 days of cultivation (Fig. 6a, b, e).

### Effect of inserting *ppc* and *pepck* on organic acid production in xylose medium

**Fig. 7:**
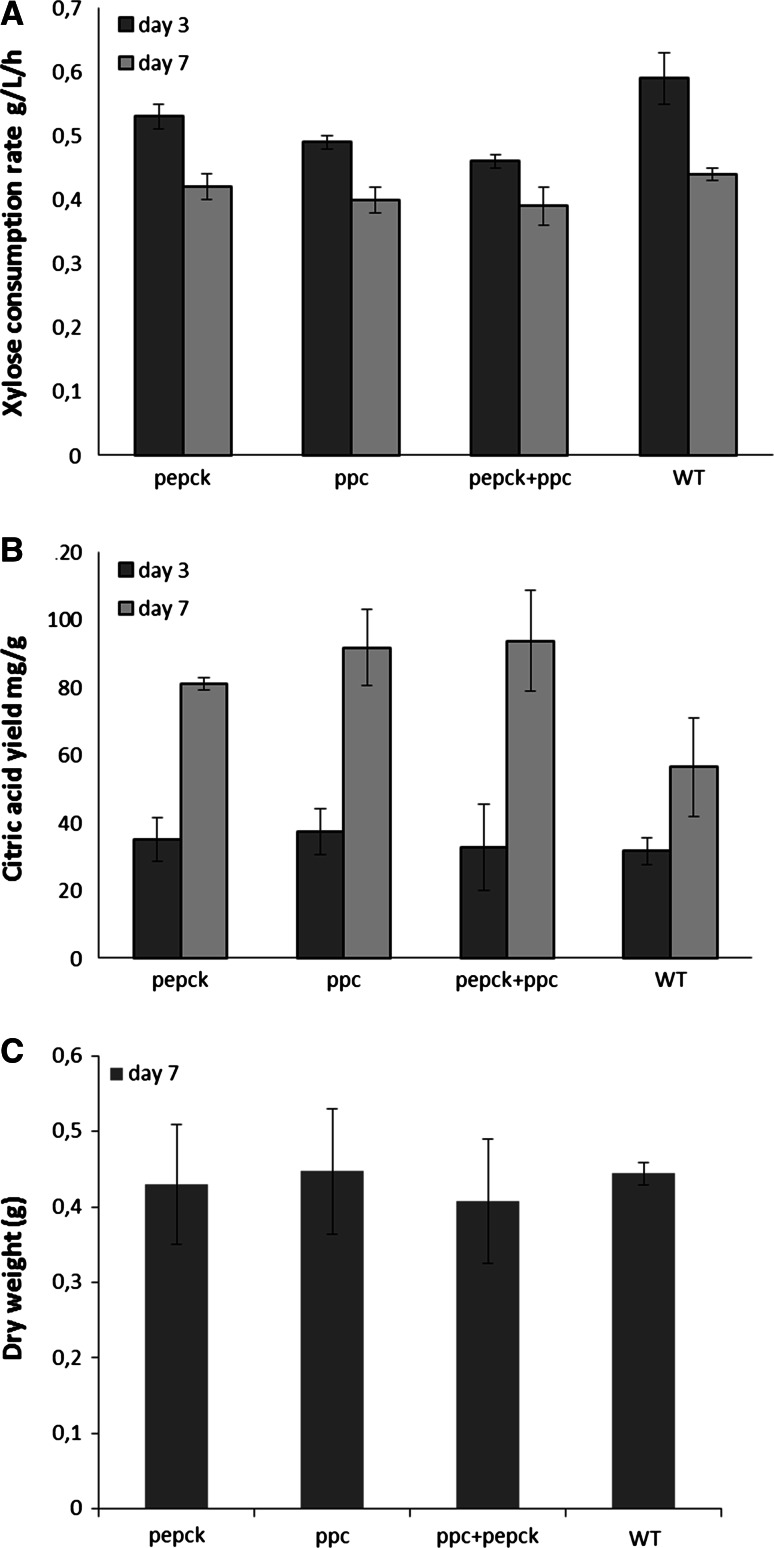
Xylose consumption rate and major extracellular organic acids in non-buffered pH xylose medium. **a** xylose consumption rate (g/L/h) by the wild type and selected transformants; **b** citric acid yield (mg/g) in the wild type strain and selected transformants; **c** dry weight (g) of fungal biomass after 7 days cultivation. (In shake flasks, 180 rpm, at 30 °C)

In order to further investigate the impact of the alternative pathway on organic acid production, the three selected transformants were also tested in xylose-based production medium. As shown in Fig. 7a, c, the genetic modifications did not significantly affect the xylose consumption rate and biomass growth in the transformants compared with the wild type. The yield of citric acid was significantly increased in all the selected transformants compared with the wild type after 7 days of cultivation (80 mg/g in *pepck* transformant, 91 mg/g in *ppc* transformant, and 93 mg/g in double transformant vs. 56 mg/g in the wild type, *p* < 0.05), although no increase in the yield of citric acid was observed on day 3 (Fig. 7b). It is also worth mentioning that, although no neutralizer was added to maintain the pH during cultivation, pH was still 3–3.5 in all the cultivation samples after 7 days, compared with pH 2–2.5 in glucose medium (Supplementary material 3).

## Discussion

In this study, two different bacterial genes (*pepck* and *ppc*) that convert phosphoenolpyruvate directly to oxaloacetate were inserted in the filamentous fungus *A. carbonarius* ITEM 5010 in order to investigate the impact on organic acid production. The derived strains were tested under different cultivation conditions to exploit their potentials for the production of different organic acids. The profile analysis of the organic acids produced by *A. carbonarius* wild type strain was conducted, and a comparison of the pattern of the different organic acids produced in non-buffered pH (2–2.5) and buffered pH 5.5 conditions revealed that the acid-producing pathways in *A. carbonarius* are influenced by ambient pH. Like *A. niger* [2, 24], *A. carbonarius* is able to produce high amount of citric acid at low pH under similar cultivation conditions, whereas at pH 5.5 which is usually adopted for producing malic acid or fumaric acid by filamentous fungi, the production of citric acid was almost inhibited, and the resulting response by *A. carbonarius* was to accumulate high amount of gluconic acid using high glucose concentration as carbon source similar to what is reported in *A. niger* [28]. It was observed that glucose was rapidly converted into gluconate by *A. carbonarius* leading to a rapid depletion of glucose in the early phase of cultivation (Fig. 2b). Although the gluconate could also be utilized by *A. carbonarius* as a carbon source, the uncertain effect of shifting carbon sources on cell metabolism might affect the productivity and amount of organic acid production.

In filamentous fungi, it has been shown that the rTCA branch is highly involved in production of different organic acids [3, 5, 17, 27, 39]. In order to investigate the impact of increasing carbon flux toward the rTCA branch for organic acid production, a new cytosolic bypass was constructed by expressing the genes *pepck* from *A.succinogenes* and *ppc* from *E. coli* in *A. carbonarius.* Two derived strains with individual insertion of the *pp*c and *pepck* genes, respectively, were first tested in non-buffered pH condition. Oxalic acid increased slightly in the culture broth of the transformants compared with the wild type, but there was no significant increase in the yield of citric acid. It seems that the impact of this cytosolic bypass is very limited on organic acid production in *A. carbonarius* in this condition. However, at pH 5.5, the yield of citric acid increased significantly in the engineered strains compared with the wild type. This result implies that the effects of the heterologous expressed genes on organic acid production are dependent on cultivation conditions. In addition to the different ambient pH, the carbon dioxide released from the neutralizer CaCO_3_ may also facilitate the PEP carboxylation since it is required as the co-substrate in the reaction.

To explain the enhanced citric acid production observed in the engineered strains, it is likely that the alternative cytosolic pathway enhanced the carbon flux toward OAA and then further increased the intracellular concentration of cytosolic C4-dicarboxylic acids which in *A. niger* has been demonstrated to positively correlate to citric acid production [3]. The competition between the inserted pathway and the original glycolytic pathway on the substrate PEP could lead to an increased carbon flux toward the reductive TCA pathway by reducing the generation of pyruvate. In eukaryotic cells, pyruvate carboxylase is normally regulated by acetyl-CoA, which may result in the strict allocation of carbon flux between OAA and acetyl-CoA [4, 14]. By introducing this alternative pathway, it provides the possibility to establish a new balance on those two key intermediates in the metabolic pathway in *A. carbonarius.* In an attempt to further enhance the carbon flux via the inserted bypass, a strain with two different genes (*ppc* and *pepck*) was constructed by transforming the wild type with two individual plasmids containing the *ppc* and *pepck* genes simultaneously. This double transformant was constructed to investigate eventual combined effects of the two genetic modifications on organic acid production. However, no further increase of citric acid production or any significant change in other organic acids was observed compared with the *ppc* and *pepck* single transformants. A similar result has been reported in *E. coli*, when AsPEPCK was heterologously expressed in *E. coli* containing the *ppc* gene. No significant increase in succinic acid production was observed in this strain [18]. In another study, a positive synergistic effect of inserting the two genes in combination was observed on succinic acid production in *E. coli* when the EcPPC activity could be controlled below a certain level since AsPEPCK has lower affinity to PEP than EcPPC and it carries out a reversible reaction of PEP to OAA [15, 20, 33]. In addition, in *A. carbonarius,* the potential competition between the inserted bypass and the original glycolytic pathway on carbon flux may also limit the impact of expressing two enzymes in combination on organic acid production. Therefore, simply expressing these two genes in combination seems not to be a suitable strategy to reroute more carbon flow toward the new cytosolic bypass from glycolysis.

The three engineered strains were also tested in a xylose medium. In a previous study carried out on *A. carbonarius* wild type, it was shown that *A. carbonarius* was able to produce citric acid from xylose [36]. As xylose is utilized through the pentose phosphate pathway in the cell and the resulting carbon flux joins into the glycolytic pathway (Fig. 1), it may also provide a similar platform to evaluate the effect of the inserted bypass on organic acid production. Under these conditions, the expression of the *ppc* and *pepck* genes also led to an enhanced citric acid production in the engineered strains, which is similar to the result obtained in the glucose medium at pH 5.5 but opposite to the results from non-buffered cultivation. Compared with the pH change in the non-buffered cultivation using glucose as substrate, it was shown that pH decreased slower in the xylose medium (Supplementary material 3). After 7 days of cultivation, the final pH measured in the culture was 3–3.5. The slow pH decrease during cultivation may influence the productivity through the inserted bypass and the relevant metabolic pathways. In *A. niger*, oxalic acid was dramatically inhibited in cultivation at pH lower than 3 compared to pH 5. It was assumed that the oxaloacetate hydrolyase was inhibited by lowering the ambient pH below 3 [29]. Therefore, the enhanced citric acid production in the xylose medium also indicates that the impact of this new bypass on organic acid production by *A. carbonarius* exists in a certain pH range.

Finally, it seems that there is no clear difference between the bypasses carried out by AsPEPCK and EcPPC. The main difference in the reactions catalyzed by PPC and PEPCK is ATP yield. In the reaction carried out by PEPCK, one ATP is generated together with one molecule OAA. However, the reaction carried out by PPC is ATP neutral, as only one phosphate group is generated together with OAA. Therefore, compared with the original cytosolic reductive pathway, the inserted cytosolic bypass with AsPEPCK is supposed to influence both the carbon flux and energy balance in *A. carbonarius*. It has been reported in genetic engineered *E. coli* that higher succinic acid production was observed in transformants overexpressing AsPEPCK than overexpressing EcPPC [23]. The differences of those two genes were also observed in their impacts on cell growth when they were expressed in a *S. cerevisiae* mutant devoid of pyruvate carboxylases. Overexpression of EcPPC in *S. cerevisiae∆pyc* mutant can restore the cell growth in glucose medium, but it was not observed in a strain overexpressing AsPEPCK [4, 38]. However, in this study, insertions of two genes individually into the wild type lead to a similar change in organic acid production in *A. carbonarius*. On the other hand, inserting this cytosolic bypass did not result in any overflow of malic acid or fumaric acid via the rTCA branch. The low amount of malic acid production observed in the transformants demonstrates that one genetic modification targeting in the onset of rTCA branch is not enough to reroute the carbon flux from the production citric acid to other organic acids like malic acid or fumaric acid.

## Conclusions

In this study, a profile analysis of organic acid production was carried out in *A. carbonarius* wild type cultivated at non-buffered pH and at pH 5.5. The pattern of organic acids as well as the amount of organic acid production was influenced by pH. An alternative cytosolic pathway has been constructed by inserting two heterologous genes *pepck* and *ppc* in *A. carbonarius*. The derived strains carrying individual and combined gene insertions were tested to verify the impact of corresponding genetic modifications on organic acid production. An enhanced production of citric acid was obtained in all the derived strains in both glucose and xylose media when cultivation pH was above 3 but not in glucose cultivation at lower pH. This study demonstrates that insertion of *ppc* and *pepck* in *A. carbonarius* increases carbon flux toward the rTCA branch resulting in increased citric acid production, and it also implies that the cultivation conditions, like ambient pH, affect the acid producing pathway. The further consideration on production process and conditions is necessary when designing strategies of metabolic engineering for specific products. A series of genetic modifications are required for improving the capability of *A. carbonarius* as a new cell factory for the production of different organic acids.

## Electronic supplementary material

Supplementary material 1 (DOCX 253 kb)
