# Supplementary material for: Effects of heterologous expression of phosphoenolpyruvate carboxykinase and phosphoenolpyruvate carboxylase on organic acid production in Aspergillus carbonarius
Source: J Ind Microbiol Biotechnol. 2015 Sep 24;42(11):1533–45. doi: 10.1007/s10295-015-1688-4 (PMC4607725; doi:10.1007/s10295-015-1688-4)
Supplement: Supplementary file 1 — Supplementary material 1 (DOCX 253 kb) [file 10295_2015_1688_MOESM1_ESM.docx]

**Supplementary materials to**

**Effects of heterologous expression of phosphoenolpyruvate carboxykinase and phosphoenolpyruvate carboxylase on organic acid production in *Aspergillus carbonarius***

Lei Yang, Mette Lübeck and Peter S. Lübeck^*^

Section for Sustainable Biotechnology, Department of Chemistry and Bioscience, Aalborg University Copenhagen, A. C. Meyers Vaenge 15, DK-2450 Copenhagen SV, Denmark

* Corresponding author PSL: [psl@bio.aau.dk](mailto:psl@bio.aau.dk)

Supplementary material 1

pH value in the glucose media under pH buffered and non-buffered conditions


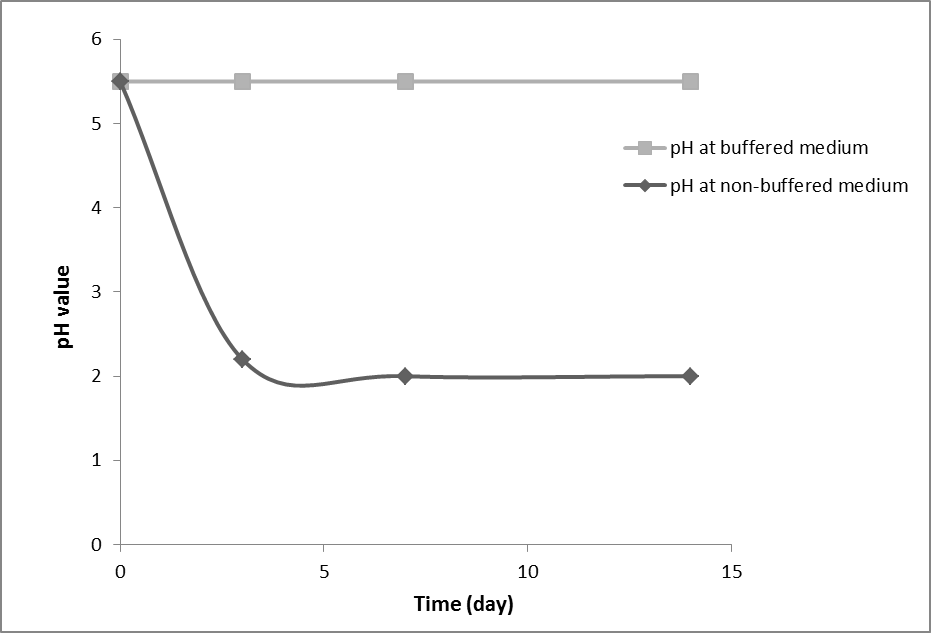


Supplementary material 2

Plasmid maps


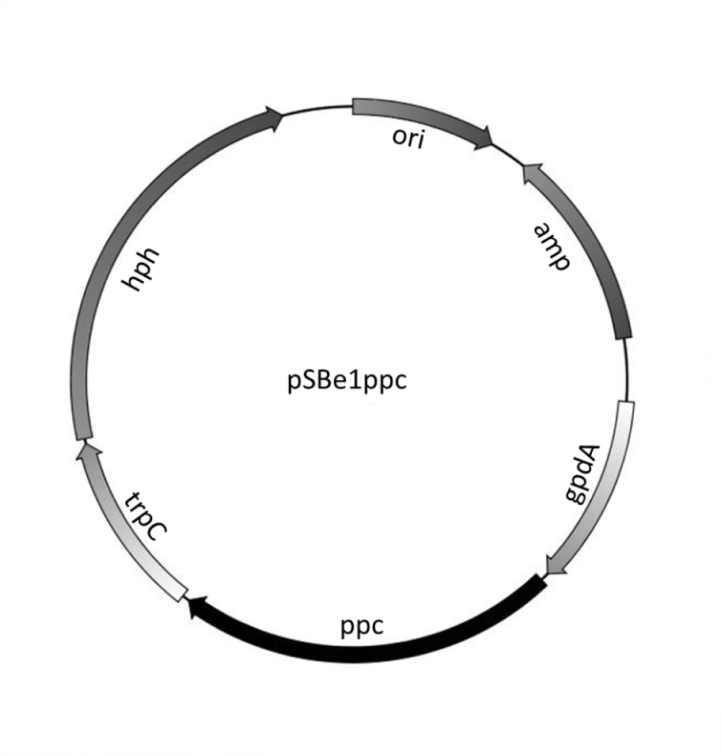


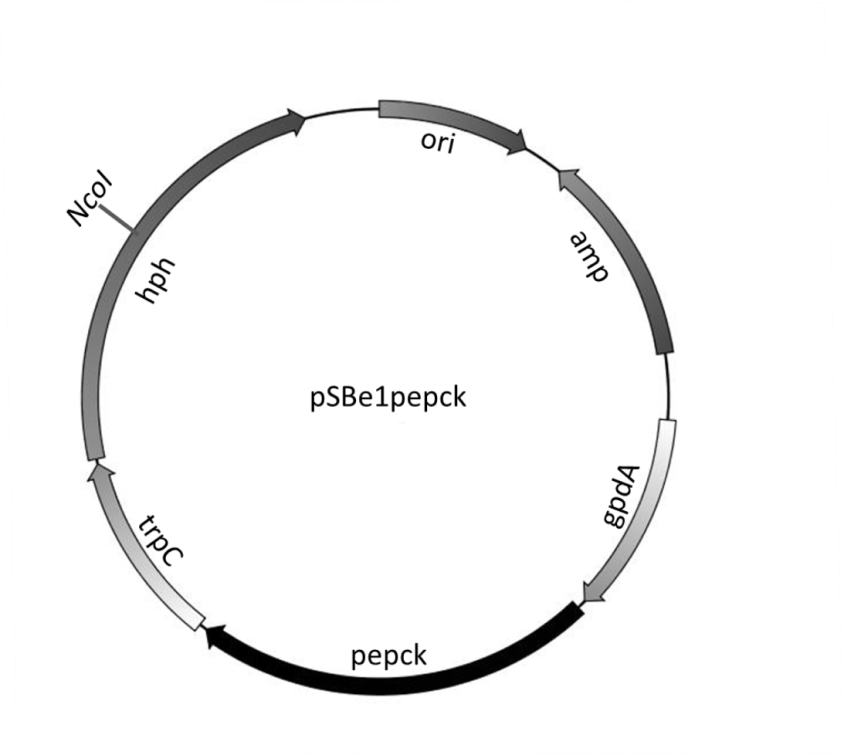


Supplementary material 3

pH values in the non-buffered pH cultures containing glucose and xylose

|  | Glucose based medium | | | | Xylose based medium | | | |
| --- | --- | --- | --- | --- | --- | --- | --- | --- |
|  | **pepck** | **ppc** | **pepck+ppc** | **WT** | **pepck** | **ppc** | **pepck+ppc** | **WT** |
| Day 3 | 2-2.5 | 2-2.5 | 2-2.5 | 2-2.5 | 3.5 | 3.5 | 3.5 | 3.5 |
| Day 7 | 2 | 2 | 2 | 2 | 3-3.5 | 3-3.5 | 3-3.5 | 3-3.5 |
